# The role of lycopene for the amelioration of glycaemic status and peripheral antioxidant capacity among the Type II diabetes mellitus patients: a case–control study

**DOI:** 10.1080/07853890.2021.1943515

**Published:** 2021-06-28

**Authors:** Hui Eng Leh, Mastura Mohd Sopian, Mohamad Hafizi Abu Bakar, Lai Kuan Lee

**Affiliations:** a Food Technology Program, School of Industrial Technology, Universiti Sains Malaysia, Gelugor, Pulau Pinang, Malaysia; b Oncology and Radiological Sciences Cluster, Advanced Medical and Dental Institute, Universiti Sains Malaysia, Bertam, Kepala Batas, Pulau Pinang, Malaysia; c Bioprocess Technology Program, School of Industrial Technology, Universiti Sains Malaysia, Gelugor, Pulau Pinang, Malaysia

**Keywords:** Antioxidant, lycopene, oxidative stress, Type II diabetes mellitus

## Abstract

**Background:**

The use of lycopene as a complementary medicine for Type II diabetes mellitus (T2DM) is limited and controversial. This study evaluated the effect of lycopene intake on the changes of glycaemic status and antioxidant capacity among the T2DM patients.

**Patients and methods:**

This case–control study involved the participation of 87 patients and 122 healthy individuals. Lycopene intake was assessed by using a food frequency questionnaire. The peripheral antioxidant capacity among the T2DM patients was evaluated. Glycated haemoglobin (HbA1c) and fasting plasma glucose (FPG) were measured as indications of glycaemic status.

**Results:**

Peripheral antioxidant capacity was significantly lower in the T2DM group. Direct positive correlations were found between the lycopene intake and peripheral antioxidant level among the T2DM patients. Contrarily, HbA1c and FPG levels decreased significantly with the higher lycopene intake.

**Conclusions:**

T2DM patients with a higher lycopene intake showed a greater peripheral antioxidant capacity and better glycaemic control. Lycopene may act to ameliorate oxidative stress and improve the pathophysiology of T2DM.

## Introduction

Type 2 diabetes mellitus (T2DM) is a complex endocrine and metabolic disorder that accounts for 90% of the diabetes cases worldwide. Within T2DM, hyperglycaemia takes place due to the impaired insulin secretion through a dysfunction of the pancreatic β-cells and insulin malfunction [1]. The International Diabetes Federation (IDF) has recorded that approximately 425 million adults were affected by diabetes, with 4 million of fatality cases annually [2]. T2DM is the most commonly found disease among the older individuals, but it has significantly affected children, adolescents and younger population lately, as a consequence of rapid urbanization, unhealthy diets and sedentary lifestyles. This growing scenario has resulted in a higher rate of obesity and diabetes, markedly in the cities of low- and middle-income countries. Undiagnosed and poorly managed diabetes would induce micro and macrovascular complications, leading to the secondary disorders, including lower limb amputation, blindness and kidney disease [3].

The pathophysiology of T2DM reveals that oxidative stress is one of the major governing factors, which attribute to insulin resistance, impaired insulin secretion, glucose utilization and impaired hepatic glucose metabolism, coupled with the activation of pro-inflammatory cytokines [4]. Persistent hyperglycaemia may increase the production of free radical *via* several pathways, notably non-enzymatic protein glycation, polyol pathway, hexosamine pathway and glucose autoxidation. Increased oxidative stress biomarkers and lower antioxidant defence have been reported among the diabetic patients, suggesting an *in vivo* overproduction of oxidizing species. In particular, diabetes patients have been featured to demonstrate a rising level of malondialdehyde (MDA) and lower level of endogenous antioxidants profiles, specifically glutathione peroxidase (GPx) and superoxide dismutase (SOD) [5].

Within this framework, lycopene, a novel phytonutrient, and a non-provitamin A carotenoid synthesized by plants and microorganisms, has attracted widespread attention for its potent antioxidative properties. Accordingly, lycopene is a lipophilic antioxidant with an open straight-chain hydrocarbon consists of mainly 11 conjugated and 2 non-conjugated double bonds, which can interrupt the chain reaction of lipid peroxidation and to quench peroxyl radicals [6]. Similarly supported by Wang et al. [7], lycopene illustrated a protective effect by the inhibition of lipogenesis and improvement of insulin resistance in the obese mice.

Recent review by Zhu et al. [8] also reported that tomato, a dominant fruit source of lycopene demonstrates a beneficial role in the management of T2DM, contributing to the lowering risk of obesity and diabetes. Such conclusion was drawn mainly with reference to the animal studies and epidemiological investigations. However, there is limited available literature pool on the detail lycopene consumption status (not only referring to tomato or tomato-based products) among the T2DM patients, while the relationship between the lycopene intake, glycaemic status and antioxidative defence mechanism among T2DM is still under investigation. In this sense, this study aims to assess the dietary intake level of lycopene, and its influence on glycaemic status, and peripheral antioxidant capacity among T2DM patients by comparing them to the age, gender and ethnicity matched control.

## Patients and methods

### Study population

A total of 87 T2DM patients were recruited from the Day Care Clinic, Advanced Medical and Dental Institute (AMDI), Universiti Sains Malaysia from June to December 2018 in Penang State, Malaysia. The inclusion criteria were listed as: (1) Chronological age of 18 years and above; (2) clinically diagnosed with T2DM for at least 6 months’ duration; (3) pharmacological treated with oral anti-diabetic agents (metformin, sulphonylureas, meglitinides, alpha-glucosidase inhibitors, thiazolidinediones, dipeptidyl peptidase-4 (DPP-4) inhibitors or sodium-glucose cotransporter 2 (SGLT2) inhibitors); and/or (4) injecting agents (glucagon-like peptide-1 (GLP-1) receptor agonists); (5) sub-optimally controlled without clinically manifest complications (retinopathy, diabetic nephropathy, foot ulcer, cardiovascular diseases, chronic kidney disease, aneurysms); (6) not taking any antioxidant or anti-inflammatory supplements. Patients with gestational diabetes mellitus (GDM), pregnancy, cancer, mental disorders, alcohol and drug abuse were excluded. Age-, gender- and ethnicity-matched healthy individuals (*n* = 122), were selected as the control group. The control group was healthy community-dwelling individual who attended the medical centre for medical check-up. The case–control matching procedure was completed based on computerized generated medical record and patient registry database. All subjects were given written consent prior to the participation in the study. The study protocol was approved by The Human Research Ethics Committee of Universiti Sains Malaysia (Approval number: USM/JEPeM/18020127), and the study has been conducted in accordance to the Declaration of Helsinki.

The following study procedures/methods were adopted:

1. Anthropometry measurements were conducted by trained nutritionist, to be recorded in a predesigned questionnaire. Body weight was measured using a digital scale to the nearest 0.1 kg, while the standing height to the nearest centimetre. A body mass index (BMI) value in the range of 18.5–22.9 kg/m^2^ is classified as normal, a BMI value between 23.0 kg/m^2^ and 32.4 kg/m^2^ is denoted as overweight, while a BMI ≥32.5 kg/m^2^ indicates an obese condition [9]. Waist and hip circumferences were taken in a standing position, and the waist to hip circumferences ratio (WHR) was calculated as an additional indicator for abdominal obesity.
2. Biochemical and blood pressure measurements including total cholesterol (TC), triglyceride (TG), low-density lipoprotein cholesterol (LDL-C) and high-density lipoprotein cholesterol (HDL-C) were conducted after an overnight fasting. Blood pressure measurement was performed using an upper arm automated blood pressure device (Omron 705IT).
3. Glycaemic status was measured as glycated haemoglobin (HbA1c) and fasting plasma glucose (FPG). Blood plasma was centrifuged from the peripheral venous blood after an overnight fasting by trained phlebotomists or staff nurses.
4. Dietary lycopene intake was assessed using a semi-quantitative food frequency questionnaire (SFFQ). The inclusion of food items containing lycopene was according to the USDA database. Eighty-three food items which are commonly consumed among the Malaysian population, and have been identified to contain high lycopene content, were classified into 12 major food groups, namely: (a) Vegetables; (b) Fruits; (c) Beverages; (d) Soup, sauces and gravies; (e) Cereal, grains and pasta; (f) Fast foods; (g) Snacks; (h) Fats and oils; (i) Legumes and legume products; (j) Finfish and shellfish products; (k) Sausages and luncheon meat and; (l) Pastry and bakery products. Raw, cooked and canned foods were included in the list. The intake frequency for each food item was evaluated according to the habitual intake over the past 12 months using three frequency intervals (per day, per week or per month). Each food item was assigned to the portion size by using the local household units: plate, bowl and tablespoon, according to the Malaysian Food Composition Table [10], and the Atlas of Food Exchanges and Portion Sizes [11]. The reference pictures of the listed food items were prepared as a printed shown document to the patients for a better serving size estimation. For those food items that are not available, the composition of 200 Foods Commonly Eaten in Singapore and nutritional food labels were consulted. Lycopene intake was computed and estimated using the Nutritionist Pro™ software (Axxya Systems LLC, Stafford, TX).
5. Assessment of peripheral antioxidant capacity was examined according to the total antioxidant capacity (TAC), GPx activity and SOD levels measurements. GPx and SOD are the first line defences of endogenous antioxidants in the body. The assessment of TAC, GPx and SOD levels were conducted using the Total Antioxidant Capacity Assay Kit, Merck Cat No. 615700; Glutathione Peroxidase Assay Kit, Merck Cat. No. 353919; Superoxide Dismutase Assay Kit II, Merck Cat. No. 574601, respectively.

### Statistical methods

The independent-sample Student’s *t* and χ^2^ tests were applied for the characteristics comparison of the T2DM patients with the control groups. The association between the lycopene intake, with the glycaemic status and peripheral antioxidant capacity was evaluated using the Pearson correlation coefficients (whole study population). The modulating effects of lycopene intake on the glycaemic status and peripheral antioxidant capacity were assessed using the generalized linear model (GLM). We first examined the effect of lycopene intake quartiles on the glycaemic control and peripheral antioxidant capacity (Model 1). The initial group of confounders (Model 2) consisted of socio-demographic variable and lipid profile. The possible confounders were included as covariates in the GLM, and Bonferroni adjustment was performed for pairwise comparisons. All descriptive data were presented as mean values ± standard deviations (SD), standard error of the mean (SEM), numbers (n) and proportions (%). All statistical tests were performed using the SPSS version 24.0 software (SPSS, Chicago, IL), with probability value less than .05 was considered as significantly different.

## Results

Two hundred and nine participants with the mean age of 56.3 ± 4.6 years, have participated in this study. The participants have been divided into two groups, namely T2DM (T2DM patients, *n* = 87) and control group (healthy adults, *n* = 122). The glycaemic status, anthropometry measurements, biochemical characteristics, lycopene intake and peripheral antioxidant capacity among the T2DM and control groups are depicted in Table 1. From the presented data, the anthropometry measurements for the two groups were similar, but the control group showed a higher HDL-C (*p*< .0001), TAC (*p*< .05), GPx (*p*< .0001) and SOD (*p*< .05) levels as compared to the T2DM group. In contrast, T2DM group demonstrated a higher FPG (*p*< .0001), HbA1c (*p*< .0001), and LDL-C (*p*< .05) levels. Meanwhile, the mean BMI for the T2DM and control group was identified at 28.5 ± 5.3 and 27.9 ± 3.3 kg/m^2^, respectively, indicating to the overweight status according to the definition of WHO Recommended BMI cut-off points for Asian population (23.0 − 32.4 kg/m^2^). Although the control group generally reported a higher dietary intake of lycopene (presumably yielded a better antioxidant capacity) as compared to the T2DM patients, the mean difference of the intake level was considered not significant.

**Table 1.** Glycaemic status, anthropometry measurements, biochemical characteristics, lycopene intake and peripheral antioxidant capacity of the T2DM and control groups.

Parameters	T2DM (*n* = 87)	Control (*n* = 122)	*p*
Glycaemic status			
FPG (mmol/L)	7.9 ± 2.7^a^	5.1 ± 1.3^b^	<.0001**
HbA1c (%)	8.0 ± 1.5^a^	4.8 ± 0.6^b^	<.0001**
Anthropometry measurements			
BMI (kg/m^2^)	28.5 ± 5.3	27.9 ± 3.3	.133
WC (cm)	98.6 ± 13.5	96.9 ± 8.2	.267
HC (cm)	104.6 ± 11.9	102.9 ± 5.8	.619
MUAC	12.5 ± 1.9	12.8 ± 0.6	.468
CC	14.7 ± 1.9	15.1 ± 0.8	.332
Biochemical characteristics			
SBP (mmHg)	141.2 ± 19.5	133.2 ± 10.3	.438
DBP (mmHg)	79.3 ± 10.8	75.8 ± 8.1	.786
TG (mmol/L)	1.8 ± 1.0	1.6 ± 0.6	.112
TC (mmol/L)	4.6 ± 1.1	4.5 ± 0.8	.133
HDL-C (mmol/L)	1.2 ± 0.3^a^	1.5 ± 0.1^b^	<.0001**
LDL-C (mmol/L)	2.7 ± 0.9^a^	2.4 ± 0.7^b^	.013*
Dietary lycopene intake (µg/d)	2759.5 ± 2307.9	2844.9 ± 2112.4	.749
Peripheral antioxidant capacity			
TAC (U/mL)	11.7 ± 1.8^a^	14.3 ± 2.9^b^	.015*
GPx (U/L)	352.7 ± 32.6^a^	398.2 ± 19.1^b^	<.0001**
SOD (U/mL)	51.1 ± 3.0^a^	63.3 ± 1.5^b^	.031*

^a,b^Values in the same row that do not share the same superscript letter are significantly different.

*p< .05; **p< .0001.

BMI: body mass index; CC: calf circumference; DBP: diastolic blood pressure; FPG: fasting plasma glucose; GPx: glutathione peroxidase; HbA1c: glycated haemoglobin; HC: hip circumference; HDL-C: high-density lipoprotein cholesterol; LDL-C: low-density lipoprotein cholesterol; MUAC: mid-upper arm circumference; SBP: systolic blood pressure; SOD: superoxide dismutase; TAC: total antioxidant capacity; TC: total cholesterol; TG: triglyceride; WC: waist circumference.

Lycopene intake by the T2DM group was inversely correlated with the FPG (*r*= −0.342, *p*<.01) and HbA1c (*r* = −0.414, *p*<.01) levels (Table 2), while lycopene intake showed positive correlation with TAC (*r* = 0.620, *p*<.0001), GPx (*r* = 0.487, *p* < .01) and SOD (*r* = 0.573, *p* < .0001) concentrations.

**Table 2.** Correlations between dietary lycopene intake, glycaemic status and peripheral antioxidant capacity.

	Correlation with dietary lycopene intake (µg/d) T2DM (*n* = 87)		Correlation with dietary lycopene intake (µg/d) Control (*n* = 122)	
	*r*	*p*	*r*	*p*
FPG (mmol/L)	−0.342	.008*	−0.112	.564
HbA1c (%)	−0.414	.004*	−0.087	.663
TAC (U/mL)	0.620	<.0001**	0.266	.149
GPx (U/L)	0.487	.002*	0.127	.233
SOD(U/mL)	0.573	<.0001**	0.098	.116

Data are presented as mean ± SD.

*p< .01, **p<.0001.

FPG: fasting plasma glucose; GPx: glutathione peroxidase; HbA1c: glycated haemoglobin; SOD: superoxide dismutase; TAC: total antioxidant capacity.

As a significant relationship was found between the dietary lycopene intake and glycaemic status, the association between HbA1c and dietary lycopene consumption quartiles was analysed. From Table 3, the HbA1c levels among the T2DM patients decreased significantly with the higher lycopene intake quartiles (*F* = 6.338, *p* < .0001). This obtained findings indicated that a lower lycopene intake yielded a poorer degree of glycaemic control among the T2DM patients. When the model was adjusted for HDL-C and LDL-C, the associations remained significant (*F* = 6.017, *p* < .0001). The TAC (*F* = 49.873, *p*<.0001), GPx (*F* = 11.204, *p* < .0001) and SOD (*F* = 18.063, *p* < .0001) values increased proportionally to the rising lycopene intake among the T2DM patients. The statistical significance was not altered after the multivariate adjustment for the covariates. The analytical results suggested that greater lycopene intake may play a pivotal role to enhance antioxidant defence mechanism for better diabetic control.

**Table 3.** Glycaemic status and peripheral antioxidant capacity among the T2DM patients by dietary lycopene quartiles.

			Lycopene intake (µg/d)				
	*F* statistic	*df*	First (<1262.28)	Second (1262.28–2142.14)	Third (2142.15–3573.85)	Fourth (>3573.85)	*p*
					HbAlc (%)		
Model 1	6.338	3	7.9 ± 0.5^a^	7.6 ± 0.2^a^	7.2 ± 0.3^b^	6.7 ± 0.1^c^	.001*
Model 2	6.017	3	8.0 ± 0.2^a^	7.5 ± 0.1^b^	7.0 ± 0.3^c^	6.4 ± 0.3^d^	<.0001**
					TAC (U/mL)		
Model 1	49.873	3	10.2 ± 0.3^a^	10.4 ± 0.2^a^	12.7 ± 0.3^b^	13.8 ± 0.3^c^	<.0001**
Model 2	47.338	3	10.2 ± 0.2^a^	11.0 ± 0.6^b^	12.9 ± 0.6^c^	14.1 ± 0.6^d^	<.0001**
					GPx (U/L)		
Model 1	11.204	3	337.3 ± 7.0^a^	335.4 ± 6.4^a^	356.7 ± 6.8^b^	385.1 ± 7.0^c^	<.0001**
Model 2	9.638	3	343.2 ± 6.8^a^	347.9 ± 4.3^a^	355.3 ± 3.2^b^	377.1 ± 5.5^c^	.001*
					SOD (U/mL)		
Model 1	18.063	3	49.0 ± 0.6^a^	49.4 ± 0.5^a^	52.2 ± 0.6^b^	54.1 ± 0.6^c^	<.0001**
Model 2	15.556	3	47.9 ± 0.4^a^	49.8 ± 0.2^b^	53.1 ± 0.3^c^	54.5 ± 0.4^d^	<.0001**

Data are presented as mean ± SEM.

^a,b,c^Values in the same row that do not share the same superscript letter are significantly different.

*p<.01, **p< .0001.

Model 1: No adjustment; Model 2: Adjusted for HDL-C and LDL-C.

GPx: glutathione peroxidase; HbA1c: glycated haemoglobin; SOD: superoxide dismutase; TAC: total antioxidant capacity.

## Discussion

The study was designed to evaluate the role of lycopene in modulating glycaemic status, and the degree of peripheral antioxidative capacity among the patients with T2DM. This study was the first within our knowledge to investigate the influence of dietary lycopene intake, instead of tomato consumption, on glycaemic control and oxidative stress. The data from this study suggested that dietary lycopene intake among the diabetic population was 0.04** **mg/kg body weight/day. The reported daily lycopene intake was insufficient according to the acceptable daily intake (ADI) of 0.05 mg/kg body weight of lycopene per day [12]. The unsatisfactory intake of lycopene may be due to the different dietary eating patterns driven by the cultural boundaries. Reported findings from the Malaysian Adults Nutrition Survey (MANS) showed that white rice, marine fish, green leafy vegetables, bread, biscuits and traditional delicacies are the routine food items among the tested populations [13]. Lycopene is a red pigment found predominantly in fruits and vegetables notably tomatoes, pink guava and grapefruits, and tomato-derived products, not limited to spaghetti, pizza, tomato juice, tomato sauce and minestrone soup.

This study proposes an inverse association between lycopene intake, FPG and HbA1c level among T2DM patients. This indicates that T2DM patients with higher lycopene consumption were more likely to be protected against the elevated FPG and HbA1c levels. Previous studies have recorded that dietary lycopene is generally associated with significant improvement of glycaemia, glucose intolerance and HbA1c levels [14–16]. Nevertheless, epidemiological investigations on the effect of lycopene against T2DM yielded controversial findings. A double-blind, placebo-controlled clinical trial demonstrated that daily lycopene consumption at 10 mg for 2 months may play a beneficial role for the prevention of long-term complications among T2DM, *via* the rising TAC levels, inhibiting the formation of MDA-LDL, and enhancement of serum immunoglobulin Ml levels [17]. Another research reported that short term supplementation of tomato juice at 500 mL/d may decrease the LDL oxidation among T2DM patients [18]. However, prospective cohort studies conducted in Finland [19] and United States [20] did not show any correlation between the dietary lycopene and incidence of T2DM. The associations between lycopene consumption and T2DM may be attenuated by several limitations. First, the disease stage of recruited diabetic patients may compromise the results, and second, the difference within these self-reported food frequency questionnaires applied for the estimation of dietary lycopene intake may be hampered by the recall bias.

TAC is the measurement of total antioxidant status with colorimetric assay analysis. GPx, an antioxidant seleno-enzyme, is responsible for the catalytic reduction of lipid hydroperoxides (ROOH and H_2_O_2_) by using glutathione as substrate [21]. SOD could catalyse the dismutation of superoxide radical (O^2−^) into H_2_O_2_ or O_2_, to serve as a defence system for cell protection against oxidative damages [22]. TAC, GPx and SOD were elected as the biomarkers for antioxidant capacity among T2DM, primarily due to the disadvantages of the commonly used assay measurements: (i) MDA is produced by the platelet enzyme thromboxane synthase during whole blood clotting and platelet activation, which can lead to excessive estimation of lipid peroxidation; and (ii) diabetes as potential confounder may enhance the levels of thiobarbituric acid reactive substances (TBARS), and more reactive α-β-unsaturated reactive aldehydes, specifically 4-hydroxy-2-nonenal (4-HNE) and acrolein [23].

An important finding of this study was the protective effect of lycopene on peripheral antioxidant levels among the T2DM patients. This could be ascertained by the fact that the greater lycopene intake level by the T2DM patients indicated the higher levels of SOD and GPx [5]. Bose and colleagues [24] also reported that a higher lycopene intake may increase the SOD and GPx level, but reduced the plasma MDA among T2DM patients. A significant increase of total antioxidant status following a high mono-unsaturated fatty acid (MUFA) + lycopene paste supplementation has been documented by [25]. The mechanisms by which how lycopene achieve its protective effect has not been well established. A proposed mechanism has suggested that lycopene may exert excellent anti-inflammatory and antioxidant properties [8]. This is attributed by its ability to regulate the AGE/RAGE, JNK/MAPK, PI3K/Akt and SIRT1/FoxO1/PPARγ signalling pathways and AchE activity. Considerable evidence has suggested that hyperglycaemia, hyperinsulinaemia and insulin resistance could result in a greater ROS production, contributing to the oxidative stress condition. In parallel, increased ROS production can impair insulin action and glucose disposal in the peripheral tissues, leading to the β-cell dysfunction and activate the progress diabetic condition, and its complications [26].

Lycopene plays an important role to maintain the redox homeostasis, and as a potent antioxidant. It reacts as a free radicals’ scavenger in preventing oxidative damage of the essential biomolecules, notably lipids, proteins and DNA. The open-chain unsaturated structure of lycopene serves as a platform to react more easily with oxygen due to its instability with weak inked form, resulting in oxidative degradation [27]. Lycopene is also believed to reduce lipid peroxidation by acting as a good chain-breaking antioxidant, in reacting with peroxyl radicals formed in the propagation phase in the formation of carbon centred radicals. These radicals can react readily and reversibly with oxygen to form new chain-carrying peroxyl radicals, which are highly stable than ROS [28]. Additionally, lycopene was capable to inhibit ROS production induced by 7-ketocholesterol in the human macrophages, directly by its antioxidant properties, or indirectly by its ability in the expression of NADPH oxidase [29]. The overall benefits may contribute to a remarkably decline in the oxidative stress damage.

This is an early study highlighted the impact of dietary consumption of lycopene on glycaemic control, and peripheral antioxidant capacity among the T2DM patients, and it bridges the gap in the available research literature. All patients were non-institutionalized, which allows direct extrapolation to other population at large. In addition, a series of potential factors (ranging from socio-demography background, health status, nutritional to clinical aspects) were included in the study, and therefore the effects of confounding variables could be eliminated. Nevertheless, there are some limitations in this study that should be acknowledged. It would be more valuable to have a peripheral reading of lycopene for better interpretation of its correlation with the glycaemic status and antioxidant status. Semi-quantitative FFQ was applied to estimate the lycopene intake, and the accuracy of the reported data was very much dependent on the subject’s ability to recall the specific foods consumed. However, the dietary method adopted for the lycopene intake estimation in the present work was valid, even for larger scale studies [30]. For a better reflection on the marginal benefits of lycopene intake, prospective clinical trials are suggested for the derivation on the relationship between the lycopene intake with the progress of T2DM. Specific considerations should be emphasized on the targeted T2DM populations, and an accurate measurement of lycopene consumption and inclusion of other confounding factors could be integrated for the corroboration of new findings.

## Conclusions

This study demonstrated that reduced peripheral antioxidant capacity occurs among the T2DM patients. Dietary lycopene intake among the diabetic population was 0.04 mg/kg BW/d, which indicating insufficient consumption level with reference to the ADI. However, T2DM patients with greater lycopene consumption pattern showed a higher level of peripheral antioxidant capacity and better glycaemic control. These observations are vital for the importance of lycopene metabolism in modulating the oxidative stress among the T2DM patients. Future studies outlining an in-depth understanding on the clinical significance of lycopene for diabetic management is suggested.

## Data Availability

The data are not publicly available due to the confidential handling of our research materials. Some of the research data in this manuscript are part of an existing big project which is underway (Project title: Elucidation of the roles of lycopene for the improvement of cardiometabolic profiles and inflammatory status in Type II diabetes mellitus, project funded by the Ministry of Higher Education, Malaysia).
